# Feasibility of implementing systematic social needs assessment for children with medical complexity

**DOI:** 10.1186/s43058-021-00237-3

**Published:** 2021-11-21

**Authors:** David Y. Ming, Kelley A. Jones, Elizabeth Sainz, Heidie Tkach, Amy Stewart, Ashley Cram, Madlyn C. Morreale, Samantha Dizon, Neal A. deJong

**Affiliations:** 1 Department of Pediatrics, DUMC, Duke University School of Medicine, Box 3352, Durham, NC 27710 USA; 2 Department of Medicine, Duke University School of Medicine, 2301 Erwin Rd, Durham, NC 27710 USA; 3 Department of Population Health Sciences, Duke University School of Medicine, 215 Morris St, Durham, NC 27701 USA; 4 Department of Pediatrics, University of North Carolina School of Medicine, 260 MacNider Building, CB#7220, Chapel Hill, NC 27599 USA; 5 University of North Carolina School of Public Health, 135 Dauer Drive, Chapel Hill, NC 27599 USA; 6 Legal Aid of North Carolina, 224 S. Dawson St, Raleigh, NC 27601 USA

**Keywords:** Children with medical complexity, Social determinants of health, Implementation strategy, Complex care

## Abstract

**Background:**

Children with medical complexity (CMC) have inter-related health and social needs; however, interventions to identify and respond to social needs have not been adapted for CMC. The objective of this study was to evaluate the feasibility of implementing social needs screening and assessment within pediatric complex care programs.

**Methods:**

We implemented systematic social needs assessment for CMC (SSNAC) at two tertiary care centers in three phases: (1) pre-implementation, (2) implementation, and (3) implementation monitoring. We utilized a multifaceted implementation package consisting of discrete implementation strategies within each phase. In phase 1, we adapted questions from evidence-informed screening tools into a 21-item SSNAC questionnaire, and we used published frameworks to inform implementation readiness and process. In phases 2–3, clinical staff deployed the SSNAC questionnaire to parents of CMC in-person or by phone as part of usual care and adapted to local clinical workflows. Staff used shared decision-making with parents and addressed identified needs by providing information about available resources, offering direct assistance, and making referrals to community agencies. Implementation outcomes included fidelity, feasibility, acceptability, and appropriateness.

**Results:**

Observations from clinical staff characterized fidelity to use of the SSNAC questionnaire, assessment template, and shared decision-making for follow-up on unmet social needs. Levels of agreement (5-point Likert scale; 1 = completely disagree; 5 = completely agree) rated by staff for key implementation outcomes were moderate to high for acceptability (mean = 4.7; range = 3–5), feasibility (mean = 4.2; range = 3–5), and appropriateness (mean = 4.6; range = 4-5). 49 SSNAC questionnaires were completed with a 91% response rate. Among participating parents, 37 (76%) reported ≥ 1 social need, including food/nutrition benefits (41%), housing (18%), and caregiver needs (29%). Staff responses included information provision (41%), direct assistance (30%), and agency referral (30%).

**Conclusions:**

It was feasible for tertiary care center-based pediatric complex care programs to implement a standardized social needs assessment for CMC to identify and address parent-reported unmet social needs.

**Supplementary Information:**

The online version contains supplementary material available at 10.1186/s43058-021-00237-3.

Contributions to the literature

- In addition to supporting the intensive medical needs and high health service use by children with medical complexity (CMC), there is growing recognition that to advance health and well-being for all CMC, social needs must be addressed.
- Screening, assessment, and follow-up of social needs is important for children generally, yet these practices have not been adapted for CMC.
- We found that evidence-informed systematic social needs screening and assessment for CMC integrated into routine care was feasible.
- Our approach offers a practical blueprint that can be adapated by other pediatric complex care programs seeking to implement social needs screening and assessment.

## Background

Children with medical complexity (CMC) are the subset of children and youth with special healthcare needs (CYSHCN) with the most intensive health needs [1]. CMC represent approximately 1% of all children, and their health-related costs are disproportionately high [2]. High health service utilization is a common consequence of the multiple chronic conditions, needs, and functional limitations (e.g., reliance on medical technology for daily living) that CMC experience [3]. Limited support available for primary care providers to coordinate care for CMC [4, 5], need for multi-specialty clinical care for each CMC [6], and high hospital utilization [1, 7] are why the locus of care for CMC is often the tertiary care center [2]: child-specific hospitals with highly specialized staff and technical expertise to which general and smaller, community-based care sites frequently refer patients with specialty needs [8]. For these reasons, a growing number of children’s hospitals have developed pediatric complex care programs to facilitate care management, improve the care experience for CMC and their families, and reduce healthcare utilization and cost [1, 9].

Because of the multiple chronic co-morbidities faced by CMC, systems of care commonly approach this population’s health needs with medically-focused interventions—e.g., scheduling specialty clinic appointments, medication reconciliation, and securing home medical supplies. While these interventions are essential aspects of care for CMC, a medically focused approach may overlook social needs that have at least as much—if not even greater—impact on the health of CMC [10]. Social need is a broad construct that can overlap with related domains, such as social drivers (or determinants) of health (SDH), adverse childhood experiences (ACEs), and parent/family health and well-being. The health of CMC is inseparable from that of their families [10] and the presence of adverse SDH—those SDH that negatively impact overall health—and/or ACEs are associated with worse health outcomes for children [11, 12].

Given the importance of SDH to the overall health of children and CMC, social needs screening and intervention represents a potential opportunity to promote better overall health and well-being [13]. However, only a small number of validated, child-specific instruments have been published [14], and definitive evidence of positive impact of SDH screening and intervention on child health outcomes has not been clearly established [15]. While positive clinical trials of social needs screening and intervention have been conducted at primary care sites with previously healthy children [12, 16, 17], there has been little published evidence to-date about adaptation of these programs to the care of CMC. Although randomized controlled trials (RCT) of SDH screening and intervention specifically for CMC have not yet been conducted, implementation science methods are valuable within pre-RCT/pilot phase studies [18].

Despite these limitations of the evidence base, understanding how to implement SDH screening and intervention is critically important because there are growing system-level pressures from policymakers and payers to do so [19, 20]. Child health systems in the United States (US) are shifting towards value-based care and payment—health care models that incentivize better quality, outcomes, and patient/family experience at lower cos t [21, 22]—and payers are beginning to mandate population-level SDH screening (e.g., North Carolina’s statewide Medicaid managed care program) [23]. In such an evolving health care landscape increasingly focused on delivery of high-value care that addresses both social and medical drivers of health, a question of pragmatic relevance to child health systems and providers is: how can the existing evidence base be leveraged to inform implementation of SDH screening and intervention for high need, high cost patients such as CMC? To address this key question and advance the field, the primary aim of this study was to evaluate the feasibility of implementing evidence-informed social needs screening and assessment within pediatric complex care programs. Due to limited prospective studies of social needs screening within the pediatric complex care literature, our secondary aim was to measure unmet social needs reported by parents of CMC.

## Methods

### Design, setting, and participants

This was a prospective, two-site feasibility study conducted between January 2019—July 2020 to implement evidence-informed systematic social needs screening and assessment within routine clinical care for CMC. The participating sites were established complex care programs for CMC with multi-specialty care based at pediatric tertiary care centers in the Southeastern US. Since their inception, the two sites have provided inpatient and outpatient care for over 300 patients to-date; one program’s clinical care was more inpatient/hospital-focused whereas the other program was more focused on the outpatient setting. Key features of both complex care programs included interdisciplinary clinical teams, care coordination, continuity of care, and direct patient care, all of which are common features of care delivery models for CMC [9]. A third complex care program participated in the development and implementation of the intervention. Since this third site did not collect implementation data, it is not included in this report.

Parents/caregivers (referred to as “parents”) of CMC already receiving care from one of the two participating complex care programs were eligible to receive the systematic social needs assessment for CMC (SSNAC) intervention. Participants included a convenience sample of parents of eligible CMC who were approached by clinical staff as part of routine clinical care. CMC criteria were site-specific and consistent with existing operational criteria used by the two participating programs to define CMC (Additional file 1).

### Components of the systematic social needs assessment for CMC (SSNAC)

Core components of the SSNAC intervention included using an evidence-informed questionnaire to screen for unmet needs, developing a structured approach for care team members to assess reported needs using shared decision-making, integration of assessment and follow-up into routine clinical care, and using multifaceted implementation strategies to facilitate overall implementation of the intervention.

### Screening Questionnaire development

The SSNAC intervention consisted of implementing social needs screening and assessment for CMC at the two participating sites. A 21-item SSNAC questionnaire was integrated into each site’s clinical workflow; items were adapted from existing social needs screening instruments (Accountable Health Communities Health-Related Social Needs Screening Tool; NC Medicaid Standardized SDH Screening Questionnaire) [19, 24]. We selected these primary source instruments because their development was informed by published literature and a review of best practices, and they are being broadly implemented by policymakers and payers at the state (NC Medicaid) and national level (Centers for Medicare & Medicaid Services), thus are highly relevant in our practice environment.

Adaptation of screening items was done by gathering direct feedback from clinical staff and parents. Each site’s clinical staff (*n* = 6; one physician, one social worker or nurse, and one care coordinator per site) reviewed the proposed survey items and suggested site-specific adaptations before reaching verbal agreement on the final 21-item questionnaire. The questionnaire was pilot tested and cognitive interviews were conducted with five parents of CMC at each site. During pilot testing, parents reported that the items, overall, were appropriate and relevant. The questionnaire took approximately 30 min to administer in-person, and parent feedback informed changes in wording for multiple screener items. This combination of parent and clinical staff feedback resulted in refinement of the SSNAC questionnaire into the final version. The SSNAC questionnaire was administered by complex care program staff to parents of CMC. Questionnaires were built into an online survey and database system (REDCap) [25] and administered in-person or via phone.

### Assessment of social needs identified by screening questions

When parents of CMC reported unmet social needs (i.e., “positive” response to ≥ 1 items in the SSNAC questionnaire), a standardized approach guided complex care program staff’s assessment and response. Assessment of reported unmet social needs was guided by a template developed in collaboration with long-standing partners from Legal Aid of North Carolina’s statewide Medical-Legal Partnership program [26]. The assessment template was tailored to correspond with SSNAC screening questions and provided staff with the following approaches for response to unmet social needs: (a) providing information to families about how to meet the social need; (b) directly assisting the family with their social need; and/or (c) making a direct referral to a community-based agency/partner with additional resources and expertise to meet the need. Specific questions prompted shared decision-making between clinical staff and parents to identify the social needs for which parents wanted immediate additional assistance.

### Implementation process

**Fig. 1:**
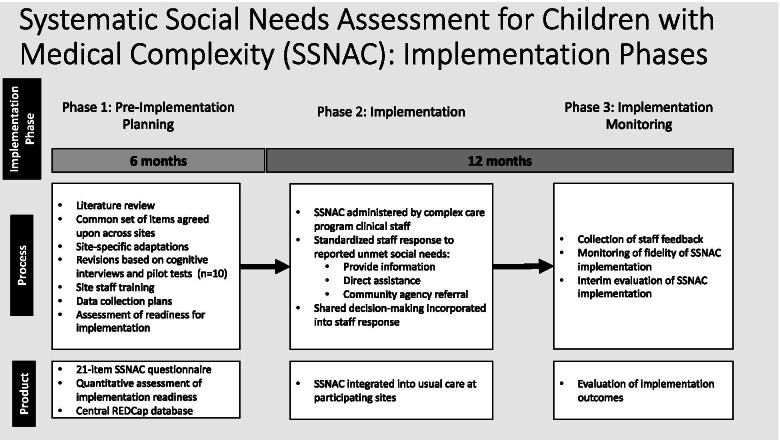
Phases of systematic social needs assessment for CMC (SSNAC)

SSNAC implementation followed three phases: (1) pre-implementation planning, (2) implementation, and (3) implementation monitoring (Fig. 1). We mapped key steps in the SSNAC implementation process to an evidence-based framework (Knowledge to Action) [27]. We utilized a multifaceted implementation package that consisted of multiple discrete, evidence-based implementation strategies [28] utilized in each project phase.

In the pre-implementation planning phase, we conducted a local needs assessment at each site consisting of assessment of implementation readiness, educational meetings with staff, and gathering feedback from parents. Prior to SSNAC implementation, multiple virtual educational planning meetings were held with staff from both sites to facilitate coalition building. SSNAC implementation readiness was measured with Hexagon Tool [29] ratings by clinical staff on a five-point Likert scale (1 = lowest; 5 = highest). Mean ratings from eight respondents across both sites in six domains of implementation readiness were: evidence [4], usability (3.8), program supports (3.4), fit (4.6), need (3.4), and capacity (3.4). We used these responses to guide development of site-based workflows for the implementation phase.

In the implementation phase, the primary implementation strategy utilized was adaptation to tailor within local context. For example, complex care program staff that deployed the SSNAC questionnaire varied by site; one site utilized a clinical social worker while the other utilized a nurse care coordinator. Another example of adaptation was that in-person social needs screenings occurred either during clinic or hospital encounters in order to align with each participating complex care program’s predominant clinical focus on outpatient or inpatient care. In-person screenings were initially the predominant approach at both sites; however, phone screenings became the primary modality subsequent to the onset of the COVID-19 pandemic in March 2020.

The implementation monitoring phase occurred concurrently with the implementation phase and continued through the end of the study. We measured fidelity by querying staff via online surveys following each SSNAC questionnaire for self-report of their use of the SSNAC questionnaire, use of the assessment template, and incorporation of shared decision-making. During this phase, we also regularly audited performance via monthly, open-ended surveys to further characterize real-world implementation from staff perspectives (e.g., usability of the questionnaire, challenges faced, parent feedback).

### Implementation outcomes

Evidence-based implementation outcomes [30] included fidelity (degree to which SSNAC questionnaire was used as planned), feasibility (extent to which social needs assessment can be successfully carried out within complex care programs), acceptability (perception among program staff that social needs assessment was agreeable and satisfactory), and appropriateness (perceived fit/relevance of social needs assessment for complex care practice settings). Fidelity was measured by the following: (1) proportion of parents approached who completed SSNAC questionnaires and (2) staff self-report of use of the SSNAC questionnaire, shared decision-making, and assessment template for each completed screening, respectively. We measured feasibility, acceptability, and appropriateness outcomes by surveying clinical staff with validated instruments (4 items each rated on a 5-point Likert scale) at the end of the study period [31].

### Analyses

Five respondents (out of 54) did not complete any social needs items and were removed to create a final sample size of 49 parent respondents. For analysis of implementation outcomes, survey responses from five clinical staff (out of six total) across the two sites were analyzed at the end of the study period to assess feasibility, acceptability, and appropriateness by calculating the mean and standard deviation per outcome. Staff responses to surveys upon completion of each SSNAC questionnaire were summarized to assess fidelity to SSNAC core components. For analysis of parent responses to the SSNAC questionnaire, reporting of unmet social needs included all participants except for items asked based on endorsement of a particular need, as noted in the corresponding table (Table 3). Responses to unmet needs were calculated for those parents who reported one or more unmet need. Descriptive statistics were calculated using proportions and frequencies for categorical variables and means and standard deviations for continuous variables. All analyses were completed using SAS v9.4. This study was classified as exempt by our institutional Internal Review Board.

## Results

### Participant characteristics

During the 12-month implementation phase, 49 CMC received the SSNAC intervention with 43% (*n* = 21) at one site and 57% (*n* = 28) at the second site. The average patient’s age was 8.6 years old; 71% were Hispanic, Black, or multiracial; and 80% were publicly insured (Table 1). On average, seven distinct specialists were involved in each child’s care and reliance on medical technology for daily living was common; the most prevalent medical devices were feeding tubes (82%), supplemental oxygen (35%), tracheostomy (22%), and ventriculoperitoneal shunts (20%). The vast majority of respondents (96%) to the SSNAC questionnaire were parents and 53% had some college or higher educational level. Fifty-six percent of parents reported household annual income of $40,000 or less, the reported parental unemployment rate was 18%, the average household size was four people, and 69% reported living in urban home communities.

**Table 1:** Patient and parent characteristics

Variable	Overall
*N*	49
**Patient demographic characteristics**	
Patient age (years), mean (SD)	8.6 (5.8)
Patient race/ethnicity	
Black	13 (26.5%)
Hispanic	20 (40.8%)
White	14 (28.6%)
Multiracial or other	2 (4.1%)
Patient gender	
Male	32 (65.3%)
Female	17 (34.7%)
Public health insurance (Medicaid or Medicare)	39 (79.6%)
**Patient clinical characteristics**	
Specialists	
Number of specialists seen in past 12 months, mean (SD)	6.9 (2)
Neurology	39 (79.6%)
Orthopedics	28 (57.1%)
Pulmonary	28 (57.1%)
Gastrointestinal	34 (69.4%)
Otolaryngology	27 (55.1%)
Pediatric general surgery	24 (49%)
Ophthalmology	21 (42.9%)
Cardiology	21 (42.9%)
Endocrinology	20 (40.8%)
Other specialist	45 (91.8%)
Medical technology needs	
Feeding tube	40 (81.6%)
Oxygen	17 (34.7%)
Tracheostomy	11 (22.4%)
VP shunt	10 (20.4%)
Ventilator	5 (10.2%)
BiPAP	4 (8.2%)
Central line	3 (6.1%)
CPAP	2 (4.1%)
Other	18 (36.7%)
**Parent/caregiver and household characteristics**	
Caregiver relationship to child	
Parent or step-parent	47 (95.9%)
Grandparent	2 (4.1%)
Caregiver age (years), mean (SD)	37.7 (10.7)
Caregiver gender	
Male	1 (2%)
Female	48 (98%)
Caregiver education	
Less than high school degree	10 (20.4%)
High school degree	13 (26.5%)
Some college or higher	26 (53.1%)
Caregiver employment status	
Employed full time	10 (20.4%)
Employed part time	5 (10.2%)
Homemaker	17 (34.7%)
Retired	3 (6.1%)
Unemployed	9 (18.4%)
Other	5 (10.2%)
Household annual income^a^ *(n = 32)*	
Less than $20,000 per year	9 (28.1%)
$20,001–$40,000 per year	9 (28.1%)
$40,001–$60,000 per year	7 (21.9%)
$60,001–$80,000 per year	4 (12.5%)
More than $80,000 pe year	3 (9.4%)
Number of household members, mean (SD)	4.0 (1.4)
Household crowding index, median (Q1, Q3)	1.3 (1.0, 1.7)
Home community rural/urban status^b^	
Urban focused	34 (69.4%)
Large/small/isolated small rural city/town focused	10 (30.6%)

^a^17 respondents did not complete the income question and were excluded from this denominator

^b^Based on the Rural Urban Commuting Area (RUCA) classification using home zip code

*Abbreviations*: *CPAP* continuous positive airway pressure, *BiPAP* bilevel positive airway pressure, *VP* ventriculoperitoneal

### Implementation outcomes

Fidelity from the parent perspective was high—98% of parents approached by program staff agreed to initiate the SSNAC questionnaire. Among the 49 parents who initiated the questionnaire, 91% completed all social needs screening items. High levels of fidelity to the core components of the SSNAC intervention were also observed from the staff perspective with clinical staff reporting use of the SSNAC questionnaire in 100% of screenings and use of shared decision-making 92% of the time to identify social needs for which additional assistance was requested (Table 2). Clinical staff used the assessment template to guide their response for 95% of parents who reported ≥ 1 unmet social need.

**Table 2:** Implementation outcomes

Variable	Overall	Site A	Site B
*N*	49	21	28
**Fidelity**; *n* (%)			
Staff use of SSNAC to identify social needs			
Yes	49 (100%)	21 (100%)	28 (100%)
Staff use of shared decision-making to assist with social needs			
Yes	45 (91.8%)	19 (90.5%)	26 (92.9%)
Not applicable, no needs identified	4 (8.2%)	2 (9.5%)	2 (7.1%)
Staff use of assessment framework to respond to unmet social needs			
Yes	26 (53.1%)	14 (66.7%)	12 (42.9%)
Not applicable, no needs identified or parent/caregiver declined assistance	23 (46.9%)	7 (33.3%)	16 (57.1%)
Feasibility*; mean (range)	4.2 (3–5)		
Acceptability*; mean (range)	4.7 (3–5)		
Appropriateness*; mean (range)	4.6 (4–5)		

*Measured by implementation survey of staff at both sites; site-specific *n*’s and values suppressed due to small cell size (*n* = 5 total)

Staff generally found SSNAC implementation to be feasible, acceptable, and appropriate. Levels of agreement (5-point Likert scale; 1 = completely disagree; 5 = completely agree) rated by staff survey responses were moderate to high for acceptability (mean = 4.7; range = 3–5), feasibility (mean = 4.2; range = 3–5), and appropriateness (mean = 4.6; range = 4–5). Additionally, open-ended feedback from clinical staff provided further insight into the SSNAC questionnaire’s acceptability to parents and overall usability and implementation. First, staff reported that the questionnaire was straightforward, the items were clear and easily understood, and overall, parents were receptive to the questionnaire. Second, in-person deployment was preferred by staff instead of phone; a conversational approach to deployment of the questionnaire increased parents’ comfort level and facilitated individualized discussions about social stressors stemming from the COVID-19 pandemic. Finally, flexible approaches by staff facilitated integration of the questionnaire within routine clinical encounters (e.g., conducting questionnaire before physician entered clinic exam room; screening patients in the hospital before discharge; using telehealth video visits).

### Social needs reported by parents

Overall, 76% of parent respondents reported ≥ 1 unmet social need (Table 3). Thirty-seven percent (*n* = 18) requested additional assistance to address reported needs. Among the 37 parents who reported at least 1 unmet social need, 41% received additional information, 30% received direct assistance, and 30% were referred to a community-based agency for additional asssistance (Table 3).

**Table 3:** Social needs reported by CMC caregivers and program follow-up responses to those needs

Variable	Overall
*N*	49
Any unmet social needs	
No	12 (24.5%)
Yes	37 (75.5%)
**Categories of unmet social need**	
Gaps in access to food and/or nutrition benefits	20 (40.8%)
Food insecurity, including formula	17 (34.7%)
Gap in federal nutritional benefits	8 (16.3%)
Housing-related concerns	9 (18.4%)
Housing insecurity	3 (6.1%)
Housing environmental safety concerns	6 (12.2%)
Gap in home utility services	4 (8.2%)
Lack of medical transportation	6 (12.2%)
Concerns about school services^a^ *(n = 35)*	8 (22.9%)
Gap in supplemental security income benefits^b^ *(n = 11)*	8 (72.7%)
Gap in home and community-based services program waiver^c^ *(n = 10)*	8 (80%)
Interpersonal safety concerns	1 (2%)
Any caregiver needs	14 (28.6%)
High caregiver burden	9 (18.4%)
Caregiver social isolation	7 (14.3%)
Urgent social needs reported	5 (10.2%)
Asked for any help with unmet social needs	18 (36.7%)
**Follow-up response by complex care programs to address social needs**^d^	
*N*	37
Provided information, referrals, or direct assistance	20 (54.1%)
Provided information to patient/family	15 (40.5%)
Referral made to community-based partner/agency	11 (29.7%)
Direct assistance to patient/family	11 (29.7%)

Sample size varies if item was only asked of a subset of participants

^a^Among children with an IEP (individualized education program) or 504 plan

^b^Among those waiting to hear back about an SSI (supplemental security income) application or if application was recently denied

^c^Among those waiting to hear back about CAP/C (Community Alternatives Program for Children is the Medicaid Home and Community-Bsaed Services waiver program in North Carolina) application or if application was recently denied

^d^Among those with at least one unmet social need

## Discussion

In this multi-site prospective study, implementation of evidence-informed social needs screening and assessment for CMC was feasible. Several observations supported implementation feasibility. First, fidelity to the core components of the SSNAC intervention was high, as evidenced by universal use of the SSNAC questionnaire to identify needs and 92% reported use of SDM by clinical staff to align assessment with parent preferences. We hypothesize that this high level of fidelity reflected high stakeholder engagement and readiness to implement, as demonstrated by high pre-implementation ratings on the Hexagon Tool analysis. Second, this favorable implementation climate was likely fostered and feasibility was strengthened by integrating the SSNAC intervention into existing complex care program clinical operations. This pragamatic approach allowed for implementation without need for new clinical staff; in turn, this allowed for the SSNAC intervention to be budget neutral and the questionnaire to be deployed by experienced clinical staff at each site with established, therapeutic patient-clinician relationships. Finally, feasibility was supported by staff open-ended feedback and responses to evidence-based quantitative measures of implementation outcomes (feasibility, acceptability, appropriateness) [31].

Discrete implementation strategies used in different project phases contributed to successful implementation. In particular, efforts during the pre-implementation phase were key. In-depth efforts to engage with staff at both sites during the pre-implementation phase via virtual educational meetings helped to establish a sense of collaboration as a local coalition. The shared goals for the project were further facilitated by gathering feedback from site staff and parents of CMC to tailor evidence-informed social needs screening items into a final SSNAC questionnaire that was acceptable for all. Working together across sites on pre-implementation planning steps help to establish a favorable baseline implementation climate with engaged and committed stakeholders. As such, the pre-implementation phase would be critical to replicate SSNAC at future implementation sites.

During the subsequent implementation phase, the favorable implementation climate likely contributed to clinical staff’s willingness to develop flexible adaptations when confronted with real-world implementation challenges—e.g., incorporating SSNAC questionnaires into busy clinical practice settings and pivoting to virtual questionnaire deployment during the COVID-19 pandemic—thus, further facilitating fidelity to the intervention over time. Flexibility to adapt and tailor the approach to each site’s staffing model—e.g., use of social worker or a nurse for questionnaire deployment—allowed for the SSNAC intervention to best fit into existing clinical workflows.

Shared decision-making (SDM) is recommended as a patient-centered approach that stresses the patient/family’s desire for additional assistance, efficiently aligns patient/family preferences with available resources and is associated with higher rates of referral to address unmet needs [32, 33]. However, few prior social needs screening interventions have specifically incorporated SDM [12]. We were unable to characterize the specific reasons why 63% of parents of CMC declined additional assistance in our study, despite reporting high prevalence of unmet social needs. Potential explanations may include (1) concurrent access to additional support via parent-parent advice-giving and informal communication networks [34]; (2) negative prior experiences accessing or working with social support programs (e.g., food pantry, respite services); and/or (3) mistrust of programs and resources based on negative experiences for many families stemming from systematic barriers to accessing care, personal discrimination, and systemic racism [35].

### Strengths

This study had several strengths. First was the real-world implementation of SSNAC at multiple sites. Flexible approaches that utilized existing clinical staff to deploy the SSNAC questionnaire within routine clinical care facilitated implementation in different contexts. Second, we incorporated evidence-based methods—e.g., implementation frameworks and outcomes measurement—that generated insights into the real-world implementation process of social needs screening and intervention [12, 36]. Finally, incorporation of a structured assessment template that included prompts for the use of SDM helped to align staff response to social needs with the priorities and preferences of parents. Though low levels of parental request for additional assistance with needs could have been a negative reflection of the SSNAC process, positive parent feedback during pilot testing of the questionnaire and favorable staff observations during the implementation phase highlighted the perceived value and relevance of SSNAC to parents. For example, several parents appreciated being asked specifically about caregiver burden and social isolation during conversations with the clinical staff. The conversational nature reported by staff when implementing the SSNAC questionnaire within routine encounters may have strengthened relationships with parents by signaling that social needs were just as important for each child’s overall health and well-being as medical needs.

### Limitations

Several limitations of this study should be acknowledged. One category of limitations stems from unique aspects of our implementation environment. Generalizability is limited because SSNAC was implemented within complex care programs that cared for CMC with higher levels of complexity than CMC in peer programs—e.g., higher prevalence of feeding tubes, supplemental oxygen, tracheostomy, and ventriculoperitoneal shunts [37]. However, as the CMC population grows and the number of complex care programs increases [1], opportunities to apply the core components of SSNAC are growing. Generalizability is also limited for clinical settings without dedicated staff who have continuity of care and long-term relationships with families. In our sites, family/clinician relationships forged over time created a foundation of mutual trust that may have made parents more comfortable with disclosing social needs. Furthermore, particularly close, long-standing working relationships with our collaborators from Legal Aid of North Carolina’s Medical-Legal Partnership (MLP) program represented a unique asset within our environment. The role of MLPs in pediatrics is well-described [38], and we encourage other sites to explore strengthening partnerships with this valuable community-based organization. Finally, SSNAC required clinical staff to deploy the questionnaire. Models of care for CMC are heterogeneous [9] and not every program has existing staff available for social needs screening and assessment. However, our experience utilizing different types of staff (e.g., nurse, social worker) based on each site’s resources and capacity coupled with published social needs screening in non-CMC populations by non-clinical community health workers [17] suggest that staffing models can be flexible.

A second category of limitations is related to methodological considerations. First, we did not measure distal process (e.g., receipt of services) or health outcomes (e.g., quality of life, parent well-being) and focused primarily on implementation outcomes. Balancing pragmatism and methodological rigor is common in social needs research; therefore, implementation science and quality improvement methods are recommended over traditional efficacy trial designs [12]. Second, we did not use a validated scale to measure staff-reported SDM and we were unable to directly measure parent perspectives on SDM. Third, though items in the SSNAC questionnaire were drawn from those described in the published literature, the final questionnaire’s items in its fully implemented form were not validated and some terms (e.g. caregiver burden) reflected a deficits-based framework [39]. Strengths-based approaches better highlight parents’ and families’ assets available to address social needs rather than highlighting their deficits via the use of terms such as “burden.” However, multi-site consensus building, pilot testing with families of CMC, and alignment with forthcoming state-level Medicaid requirements for SDH screening [24] increased the usability and relevance of the final selected SSNAC items. Given the wide range of SDH screening instruments that are currently being used by pediatric providers, many of which are not validated [33], the specific survey selected arguably may be less important than building capacity to facilitate parent/family engagement, shared decision-making, and follow-up on identified needs [32].

### Future directions

Future research can build on these findings by implementing systematic social needs screening and assessment at additional clinical sites in order to understand (1) how to adapt the intervention’s core components for varied local contexts, (2) comparative effectiveness of different strategies for questionnaire implementation (e.g., in-person vs phone vs telehealth), (3) cost-effectiveness of various approaches, and (4) impacts on downstream health outcomes. Given the high prevalence of social needs among CMC and growing recognition of the importance of the intersection between medical and social needs by patients, providers, health systems, and payers, it will be critical to augment existing complex care models to better address social needs as a mechanism to enhance long-term health and well-being.

## Conclusion

It was feasible for pediatric complex care programs to implement systematic social needs screening and assessment to identify unmet social needs among CMC. A similar intervention can be considered for adaptation and implementation by other programs that care for CMC. Doing so can further demonstrate how to incorporate implementation science methods and clinical care in order to address social needs and advance health for vulnerable child populations.

## Supplementary Information

**Additional file 1.** Criteria to define children with medical complexity (CMC).

## Data Availability

The datasets used and/or analyzed during the current study are available from the corresponding author on reasonable request.

## Abbreviations

SSNAC: Systematic social needs assessment for children with medical complexity
SDH: Cocial drivers (determinants) of health
CMC: Children with medical complexity
CYSHCN: Children and youth with special healthcare needs
EHR: Electronic health record
SDM: Shared decision-making
NC: North Carolina
