# Supplementary material for: Feasibility of implementing systematic social needs assessment for children with medical complexity
Source: Implement Sci Commun. 2021 Nov 21;2:130. doi: 10.1186/s43058-021-00237-3 (PMC8606226; doi:10.1186/s43058-021-00237-3)
Supplement: Supplementary file 1 — Additional file 1. Criteria to define children with medical complexity (CMC). [file 43058_2021_237_MOESM1_ESM.docx]

**Additional file 1: Criteria to define children with medical complexity (CMC)**

| **Criterion*** | **Site A** | **Site B** |
| --- | --- | --- |
| Age | 0 – 18 years old | 0 – 21 years old |
| Multiple chronic conditions | N/A | Yes: chronic conditions involving ≥2 body systems |
| Consequences of multiple chronic conditions | Yes: positive response to CSHCN Screener^©^ question indicating “need for more care than usual” and positive responses to 3 of 4 of the remaining CSHCN Screener^©^ questions | N/A |
| Number of specialists seen/visits completed | Seen by ≥3 subspecialists in the past 12 months | ≥2 distinct UNC clinic visits in the past 12 months |
| Functional limitations | Long-term need for ≥1 of the following medical technology/devices: ventriculo-peritoneal (VP) shunt, long-term enteral feeding tube (e.g., G-tube, J-tube, GJ, etc.), long-term central venous catheter (e.g., port), tracheostomy, or mechanical ventilation-dependent (traditional ventilator or noninvasive positive pressure ventilation) | Long-term need for ≥1 of the following medical technology/devices: gastric feeding tube, tracheostomy, VP shunt  And/or  Non-ambulatory |
| High acute care utilization | ≥6 ED visits, ≥2 admissions, or ≥1 intensive care unit admission | ≥1 admission or ED visit |

**All site-specific criteria must be met to be defined as a CMC by each complex care program.*
